# Effect of a Weight Loss and Lifestyle Intervention on Dietary Behavior in Men with Obstructive Sleep Apnea: The INTERAPNEA Trial

**DOI:** 10.3390/nu14132731

**Published:** 2022-06-30

**Authors:** Almudena Carneiro-Barrera, Francisco J. Amaro-Gahete, Lucas Jurado-Fasoli, Germán Sáez-Roca, Carlos Martín-Carrasco, Francisco J. Tinahones, Jonatan R. Ruiz

**Affiliations:** 1 Sleep and Health Promotion Laboratory, Mind, Brain and Behavior Research Centre (CIMCYC), University of Granada, 18011 Granada, Spain; 2 Promoting Fitness and Health through Physical Activity Research Group (PROFITH), Sport and Health University Research Institute (iMUDS), Department of Physical Education and Sports, Faculty of Sport Sciences, University of Granada, 18071 Granada, Spain; amarof@ugr.es (F.J.A.-G.); juradofasoli@ugr.es (L.J.-F.); 3 EFFECTS-262 Research Group, Department of Medical Physiology, School of Medicine, University of Granada, 18071 Granada, Spain; 4 Unidad de Trastornos Respiratorios del Sueño, Servicio de Neumología, Hospital Universitario Virgen de las Nieves, 18014 Granada, Spain; gsaezroca@gmail.com (G.S.-R.); cmartincarrasco@gmail.com (C.M.-C.); 5 Centro de Investigación Biomédica en Red Fisiopatología de la Obesidad y la Nutrición (CIBEROBN), Institute of Health Carlos III, 28029 Madrid, Spain; fjtinahones@uma.es; 6 Department of Endocrinology, Virgen de la Victoria Hospital, Instituto de Investigación Biomédica de Málaga (IBIMA), University of Málaga, 29590 Málaga, Spain; 7 Instituto de Investigación Biosanitaria, ibs.Granada, 18012 Granada, Spain

**Keywords:** obstructive sleep apnea, obesity, weight loss, lifestyle intervention, dietary behavior, Mediterranean diet

## Abstract

This study investigated the effects of an eight-week interdisciplinary weight loss and lifestyle intervention on dietary behavior in men who were overweight/had obesity and moderate-to-severe obstructive sleep apnea (OSA). It was based on data from INTERAPNEA (ClinicalTrials.gov ID: NCT03851653); a randomized clinical trial conducted from April 2019 to April 2020. Men aged 18–65 years with moderate-to-severe OSA and a body mass index ≥25 kg/m^2^ were randomly assigned to a usual-care group or an eight-week interdisciplinary weight loss and lifestyle intervention combined with usual-care. Dietary behavior was assessed through the Food Behavior Checklist (FBC) and the Mediterranean Diet Adherence Screener (MEDAS). Of the 89 participants who underwent randomization, 75 completed the intervention endpoint assessment, 89 participants being therefore included in the intention-to-treat analyses, and 75 in the per-protocol approach. As compared with usual-care, the intervention group had greater improvements at intervention endpoint in dietary behavior, as measured by the FBC total score (20% increase in FBC total score, mean between-group difference, 8.7; 95% confidence interval, 5.7 to 11.7), and MEDAS total score (33% increase in MEDAS total score, mean between-group difference, 2.1; 95% CI 1.3 to 2.9). At 6 months after intervention, the intervention group also had greater improvements in both the FBC total score (15% increase) and MEDAS total score (25% increase), with mean between-group differences of 7.7 (CI 95%, 4.4 to 10.9) and 1.7 (CI 95%, 0.9 to 2.6), respectively. An eight-week interdisciplinary weight loss and lifestyle intervention resulted in meaningful and sustainable improvements in dietary behavior, including adherence to the Mediterranean diet in men who were overweight/ had obesity and CPAP-treated moderate-to-severe OSA.

## 1. Introduction

Obstructive sleep apnea (OSA), characterized by episodic upper-airway obstructions during sleep, affects nearly a billion adults aged 30–69 years globally [1] and is strongly associated with neurocognitive impairment, diminished quality of life, and an increased likelihood of hypertension, cancer, and metabolic, cardiovascular, and cerebrovascular diseases [2,3,4,5,6,7,8,9,10]. Although continuous positive airway pressure (CPAP) is the first-line treatment for OSA—a device serving to reduce upper-airway collapse during sleep [11]—CPAP adherence rates are suboptimal [12], and long-term benefits beyond reduction of apnea-hypopnea events per hour of sleep (i.e., the apnea-hypopnea index; AHI) and other indexes and parameters remain uncertain [13,14,15].

Given the complex and reciprocal interaction between OSA and obesity [16], weight loss and lifestyle interventions, including dietary change and exercise, are strongly recommended [17,18] and appears to significantly improve OSA severity, cardiometabolic comorbidities, and, thus, health-related quality of life [19,20,21,22,23,24]. However, most previous studies in this regard only include calorie-restricted diets [25], which may not be the most efficient approach for long-lasting and sustainable dietary behavior change [26,27]. Instead, alternative approaches, such as Mediterranean diets and other dietary strategies focusing on nutritional education and behavior change, have been proposed as potential strategies of choice for OSA management [26,27,28]. Still, although effective at improving dietary behaviors in persons with and without cardiovascular risk factors and other conditions [29,30,31,32,33], there is no evidence to date on the effects of this approach on the unhealthy dietary behaviors and poor quality of the diet commonly found in patients with OSA [34,35,36]. Similarly, and consecutively, there is no study investigating the association of changes in dietary behaviors and changes in OSA severity and related outcomes, such as body weight and composition in adults with OSA and who are overweight/have obesity.

The Interdisciplinary Weight Loss and Lifestyle Intervention (INTERAPNEA) trial is an open-label, parallel-group, randomized controlled trial aimed at testing the efficacy of an eight-week weight loss and lifestyle intervention for the improvement of OSA severity, body weight and composition, and cardiometabolic comorbidities in men with OSA and who are overweight/have obesity [37]. This study included two groups: a usual-care/control group, which received CPAP as the standard care of OSA, and a weight loss and lifestyle intervention group, which received an eight-week interdisciplinary intervention, including nutritional behavior change, aerobic exercise, sleep hygiene, and alcohol and tobacco cessation, combined with usual-care. Changes in OSA severity and body weight at 6 months after intervention indicated that participants in the intervention group reduced a significantly greater amount of their initial AHI and body weight (57% and 7%, respectively) than those in the usual-care/control group (2% and 1%) [20]. INTERAPNEA provides an opportunity to examine the effects of a behavior-induced weight loss intervention on dietary behavior and diet quality in men with moderate-to-severe OSA who received, among other intervention components, nutritional education and behavior change.

The aim of this study was therefore to examine the effects of an eight-week interdisciplinary weight loss and lifestyle intervention, as compared with usual-care (i.e., CPAP), on dietary behavior in men with CPAP-treated moderate-to-severe OSA and who were overweight/had obesity. Additionally, we pursued to investigate the associations of changes in dietary behavior and adherence to the Mediterranean diet with changes in OSA severity and body weight and composition outcomes. Thus, this study is the first to investigate the beneficial effects of this approach on dietary behavior and, in turn, the association of changes in this outcome with changes in OSA severity and body weight and composition in men with moderate-to-severe OSA. We hypothesized that the intervention group would have greater sustainable improvements in these dietary outcomes than the control group. Similarly, we expected that changes in dietary behavior and adherence to the Mediterranean diet would be significantly associated with changes in OSA severity and body weight and composition outcomes.

## 2. Materials and Methods

### 2.1. Study Design

The present work is an ancillary study of the INTERAPNEA randomized clinical trial [37], conducted from April 2019 to October 2020. Detailed information on the study rationale, design, and methodology has previously been published [37]. This trial is in compliance with the Consolidated Standards of Reporting Trials (CONSORT), was approved by the Clinical Research Ethics Committees of the University of Granada (Granada, Spain), Virgen de las Nieves University Hospital (Granada, Spain), and Junta de Andalucía (Spain) (0770-N-19), and is registered in the National Institutes of Health database (ClinicalTrials.gov NCT03851653).

### 2.2. Participants

**Figure 1:**
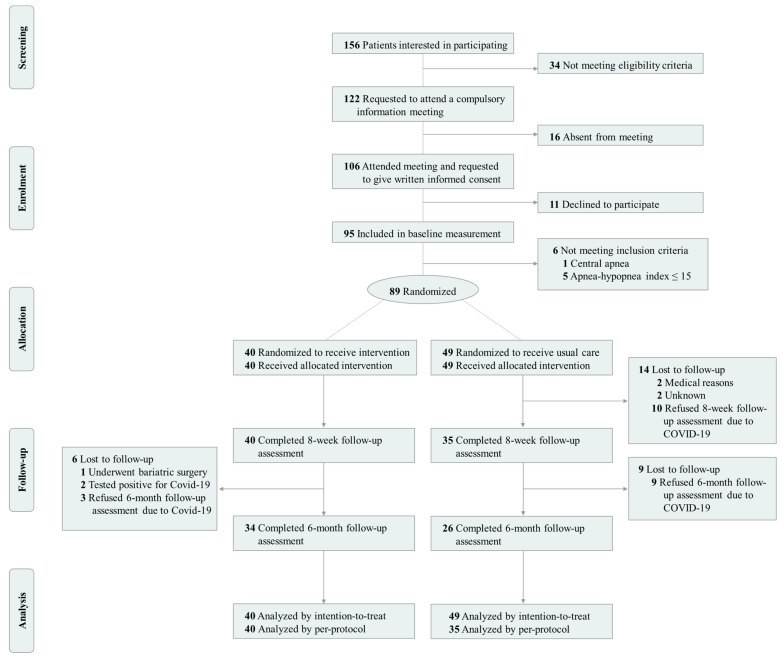
Flow-chart diagram of the INTERAPNEA randomized clinical trial.

Participants were recruited from the sleep-disordered breathing unit of the collaborating hospital (Virgen de las Nieves University Hospital). Potential participants were men aged 18–65 years with CPAP-treated moderate-to-severe OSA (i.e., AHI equal or greater than 15 events per hour of sleep) and a body mass index (BMI) equal to or greater than 25 kg/m^2^. The sole inclusion of men in our sample was not only based on the higher incidence and prevalence of OSA in this population [1], but also on the well-evidenced differences between men and women in OSA phenotypes [38] and the effectiveness of weight loss interventions [20,37,39,40,41]. Exclusion criteria included present participation in a weight loss program, presence of any psychological/psychiatric disorder, and/or any other primary sleep disorder which was not secondary to OSA. Upon providing written informed consent, potential participants were clinically/physically examined to ensure feasibility of inclusion in the study. Successively, screening/baseline measurements, including an overnight fasting blood test, complete full-night polysomnography, a set of questionnaires measuring subjective variables, and measurements of body composition and anthropometric parameters, were conducted on each participant. The trial was conducted in three consecutive sets of ~30 participants. A total of 89 men were finally randomly assigned to either the intervention group (40 participants) or the usual-care group (49 participants) by means of a computer-generated simple (unrestricted) randomization (Figure 1). The 8-week assessment (intervention endpoint) was completed by 75 participants, 15.7% (14 participants) being lost at follow-up, mainly due to the COVID-19 pandemic (10 participants).

### 2.3. Interventions

The interdisciplinary weight loss and lifestyle intervention was precisely designed following the latest clinical practice guidelines for OSA [17,18] and obesity management [42,43]. It lasted eight weeks and was composed of five components: nutritional behavior change; moderate-intensity aerobic exercise; smoking cessation; alcohol avoidance; and sleep hygiene. Participants received 60–90 min sessions weekly per component, each session being led by qualified personnel in the field (i.e., human nutrition and dietetics, physical activity and sport sciences, psychology, and sleep medicine). Briefly, the cornerstone of this interdisciplinary intervention was the use of the Transtheoretical Model of Health Behavior Change [44], a well-recognized biopsychosocial model based on integrating key strategies, processes, and principles of behavior change theories into a comprehensive interventional approach for the achievement of sustainable health-related behaviors. Consciousness raising, self-reevaluation, stimulus control, goal-setting, self-monitoring, and self-efficacy were some of the behavioral change processes and strategies used. Details of the content of the intervention have previously been published [37].

The usual-care/control group received CPAP therapy, together with a single session of 30 min addressing general advice on weight loss and lifestyle change. Nevertheless, the weight loss and lifestyle intervention was offered to all participants from this group at trial completion.

### 2.4. Assessments

Assessments at baseline, intervention endpoint, and 6 months after intervention were completed over a one to two-week period, including a fasting blood test, a full-night ambulatory polysomnography, a set of questionnaires, and a full-body dual energy X-ray absorptiometry (DXA) scanner. All participants were instructed to refrain from using CPAP for 7 days before each study assessment. A complete description of the INTERAPNEA trial assessments, including detailed information on the polysomnography performed and other tests and measures, can be found in a previously published paper [37].

The primary outcomes of this study were changes from baseline to intervention endpoint and 6 months after intervention in self-reported dietary behavior, as measured by the Food Behavior Checklist (FBC) questionnaire [45], and adherence to the Mediterranean diet, which was assessed through the Mediterranean Diet Adherence Screener (MEDAS) [46].

The FBC is composed by 22 items and seven sub-scales related to fruit and vegetables consumption (nine items), milk/dairy consumption (two items), food security (one item), diet quality (four items), fast food consumption (three items), sweetened beverages consumption (two items), and meat consumption (one item). Scores range from 23 to 85, higher scores indicating healthier dietary behavior.

Similarly, the MEDAS is a widely used 14-item screener to assess adherence to the Mediterranean dietary pattern through questions related to food intake habits and food consumption frequency. Total scores range from 1 to 14, higher scores indicating greater compliance with the Mediterranean diet. Scores equal or greater than 10 indicate high adherence to the Mediterranean diet.

Objective sleep outcomes included in this study were AHI, defined as the number of apnea and hypopnea episodes per hour of sleep, oxygen desaturation index, which is the number of oxygen desaturation ≥3% per hour of sleep, and sleep efficiency (%), calculated as the ratio of total sleep time to total time in bed. These sleep outcomes were measured through a full-night in-laboratory polysomnography.

Body weight and composition outcomes included body weight (kg), which was measured with a calibrated scale and stadiometer (model 799, Electronic Column Scale, Hamburg, Germany); neck, chest, and waist circumferences (cm); and fat mass (kg) and visceral adipose tissue (g), which were measured through a full-body DXA scanner (Discovery Wi, Hologic, Inc., Bedford, MA, USA).

### 2.5. Statistical Analysis

Linear mixed-effects models [47] were used in order to estimate intervention effects on the study outcomes. These models included group, assessment time, and their interaction terms, estimations being conducted through the restricted maximum-likelihood method and an unstructured covariance matrix adjusting for within-participant clustering resulting from the repeated-measures design. All values presented in the tables are model-based estimates, this model assuming that missing values were missing-at-random. Nevertheless, a logistic model predicting attrition propensity based on baseline values of set of participants, trial group, OSA severity, age, and BMI was used. Only set of participants predicted attrition due to the occurrence of the COVID-19 pandemic at the trial endpoint, which was the intervention endpoint assessment of the third set of participants.

Analyses and estimations were performed with both an intention-to-treat approach (including all participants as originally allocated after randomization) and a per-protocol approach restricted to participants with a CPAP usage equal or greater than four hours per night on 70% of nights and, regarding the intervention group, at least 80% of attendance rate at intervention sessions.

In addition, association of changes in dietary behavior over time with changes in sleep and body weight and composition outcomes were examined by repeated measures correlation analysis—a statistical technique used to determine the within-individual association for paired measures assessed on two or more occasions for multiple individuals [48]. All analyses were performed using R version 4.0.3 (R Project for Statistical Computing; Boston, MA, US).

## 3. Results

### 3.1. Participants’ Characteristics

Baseline characteristics of the study participants by group are presented in Table 1. The majority of participants enrolled were middle-aged men (mean ± SD age, 54.1 ± 8.0 years), with severe OSA (mean ± SD AHI, 41.3 ± 22.2 events/h) and obesity (mean ± SD BMI, 34.4 ± 5.4 kg/m^2^). There were no significant between-group differences in any of the baseline values.

### 3.2. Changes in Dietary Behavior

**Figure 2:**
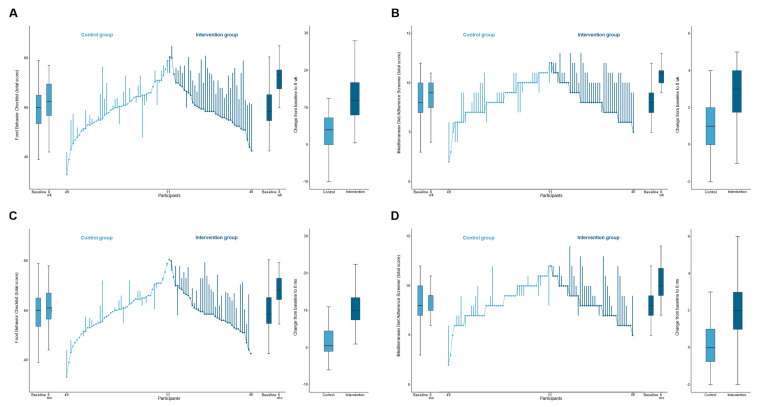
Dietary behavior outcomes. The ends of the boxes in the boxplots are located at the first and third quartiles, with the black line in the middle illustrating the median. Whiskers extend to the upper and lower adjacent values, the location of the furthest point within a distance of 1.5 interquartile ranges from the first and third quartiles. The parallel line plot contains 1 vertical line for each patient, which extends from their baseline value to their 8-week value (**A**,**B**) or 6-month value (**C**,**D**). Ascending lines indicate an improvement in the outcome. Baseline values are placed in ascending order for the control group and descending order for the intervention group. (**A**,**C**), The Food Behavior Checklist assesses dietary behavior (range, 23–85; higher scores indicate healthier dietary behavior) [45]. (**B**,**D**), The Mediterranean Diet Adherence Screener assesses adherence to the Mediterranean diet (range, 0–14; higher scores indicate greater adherence; scores ≥ 10 indicate high adherence to the Mediterranean diet) [46].

There was a significantly improvement in dietary behavior in the intervention group than in the control group, with a mean between-group difference in FBC total score of 8.7 (95% CI, −5.7 to −11.7; *p* < 0.001) and 7.7 (95% CI, −4.4 to 10.9; *p* < 0.001) from baseline to intervention endpoint and 6 months after intervention, respectively (Table 2 and Figure 2). Correspondingly, participants in the intervention group also significantly reduced consumption of sweetened beverages (*p* < 0.05), increased consumption of fruits and vegetables (*p* < 0.001), and, thus, enhanced diet quality (*p* < 0.001) (FBC subscales). Similar results were obtained using the per-protocol approach (Table S1). According to changes from intervention endpoint to 6 months after intervention, participants in the intervention group preserved improvements in these dietary behavior and diet quality outcomes, although a slight reduction in fruits and vegetables consumption was found (Table S2 and Figure S1).

Regarding adherence to the Mediterranean diet, participants in the intervention group also had a greater increase in the compliance with the Mediterranean diet, as measured by MEDAS at both intervention endpoint and 6 months after intervention, with mean between-group differences of 2.1 (95% CI, 1.3 to 2.9; *p* < 0.001) and 1.7 (95% CI, 0.9 to 2.6; *p* < 0.001), respectively (Table 2 and Figure 2). Similar results were obtained using the per-protocol approach (Table S1). Improvements in the adherence to the Mediterranean diet at the intervention endpoint were maintained 6 months after intervention (Table S2 and Figure S1).

### 3.3. Association of Changes in Dietary Behavior over Time with Changes in Sleep and Body Weight and Composition Outcomes

**Figure 3:**
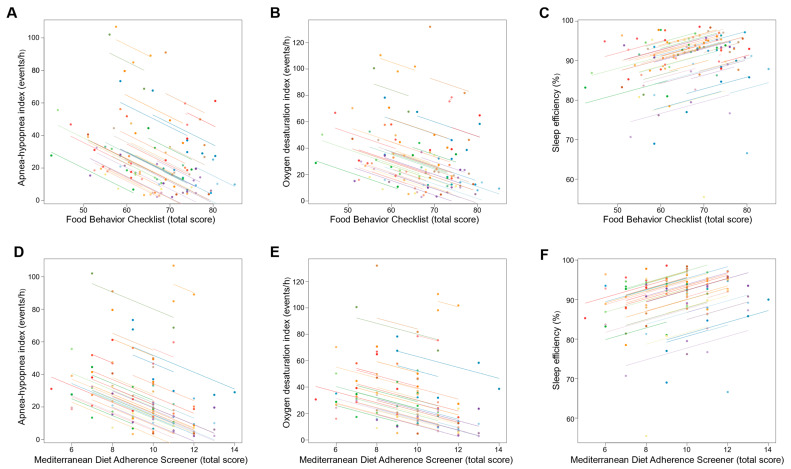
Association of changes in dietary behavior over time with changes in sleep outcomes. Each dot represents one of three separate observations (baseline, 8 weeks and 6 months after intervention) of dietary behavior—as measured by the Food Behavior Checklist (**A**–**C**) and Mediterranean Diet Adherence Screener (**D**–**F**)—and sleep outcomes for a participant. Observations from the same participant are given the same color, with corresponding lines to show the repeated measures correlation fit for each participant. (**A**–**C**) The Food Behavior Checklist assesses dietary behavior (range, 23–85; higher scores indicate healthier dietary behavior) [45]. (**D**–**F**) The Mediterranean Diet Adherence Screener assesses adherence to the Mediterranean diet (range, 0–14; higher scores indicate greater adherence; scores ≥ 10 indicate high adherence to the Mediterranean diet) [46].

**Figure 4:**
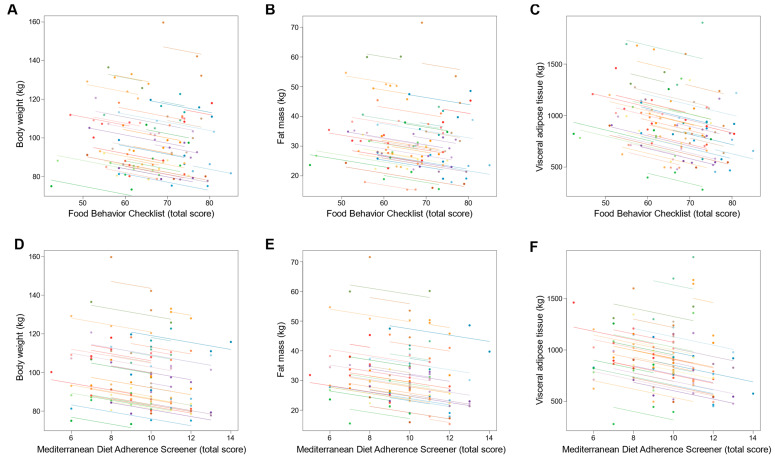
Association of changes in dietary behavior over time with changes in body weight and composition outcomes. Each dot represents one of three separate observations (baseline, 8 weeks and 6 months after intervention) of dietary behavior—as measured by the Food Behavior Checklist (**A**–**C**) and Mediterranean Diet Adherence Screener (**D**–**F**)—and body weight and composition outcomes for a participant. Observations from the same participant are given the same color, with corresponding lines to show the repeated measures correlation fit for each participant. (**A**–**C**) The Food Behavior Checklist assesses dietary behavior (range, 23–85; higher scores indicate healthier dietary behavior) [45]. (**D**–**F**) The Mediterranean Diet Adherence Screener assesses adherence to the Mediterranean diet (range, 0–14; higher scores indicate greater adherence; scores ≥ 10 indicate high adherence to the Mediterranean diet) [46].

**Figure 5:**
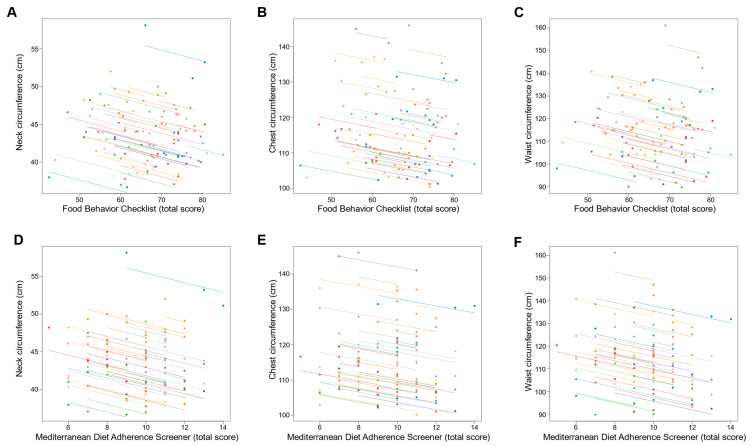
Association of changes in dietary behavior over time with changes in body circumferences outcomes Each dot represents one of three separate observations (baseline, 8 weeks and 6 months after intervention) of dietary behavior—as measured by the Food Behavior Checklist (**A**–**C**) and Mediterranean Diet Adherence Screener (**D**–**F**)—and body circumferences outcomes for a participant. Observations from the same participant are given the same color, with corresponding lines to show the repeated measures correlation fit for each participant. (**A**–**C**) The Food Behavior Checklist assesses dietary behavior (range, 23–85; higher scores indicate healthier dietary behavior) [45]. (**D**–**F**) The Mediterranean Diet Adherence Screener assesses adherence to the Mediterranean diet (range, 0–14; higher scores indicate greater adherence; scores ≥ 10 indicate high adherence to the Mediterranean diet) [46].

Changes in dietary behavior over time as measured by FBC total score were significantly associated with changes in sleep outcomes; an increase in FBC total score being related with reduced AHI and oxygen desaturation index and increased sleep efficiency (all *p* ≤ 0.001; Table 3 and Figure 3). With regards to body composition and anthropometric outcomes, changes over time in FBC total score were inversely associated with changes in body weight, fat mass, visceral adipose tissue, and neck, chest, and waist circumferences (all *p* < 0.001; Table 3 and Figure 4 and Figure 5).

Similarly, changes over time in adherence to the Mediterranean diet as measured by MEDAS total score were significantly associated with changes in sleep outcomes, an increase in MEDAS total score being related to reduced AHI and oxygen desaturation index and increased sleep efficiency (all *p* < 0.001; Table 3 and Figure 3). Increases over time in MEDAS total score were also associated with reductions in body weight, fat mass, visceral adipose tissue, and neck, chest, and waist circumferences (all *p* < 0.001; Table 3 and Figure 4 and Figure 5).

## 4. Discussion

The present study demonstrates that an eight-week interdisciplinary weight loss and lifestyle intervention, incorporating not only a nutritional behavior change component but also increased physical activity, sleep hygiene, and alcohol and tobacco avoidance is effective at significantly improving dietary behavior in men with CPAP-treated moderate-to-severe OSA and overweight/obesity. According to the results reported herein, the weight loss and lifestyle intervention group had 20% and 15% increases in healthful dietary behavior as measured by FBC at intervention endpoint and 6 months after intervention, respectively. Similarly, participants from this group reported 33% and 25% increases at intervention endpoint and 6 months after intervention, respectively, in adherence to the Mediterranean diet, as measured by MEDAS. Importantly, these improvements in dietary behavior and increases in adherence to the Mediterranean diet over time were closely related to improvements in sleep, body composition, and anthropometric outcomes.

These results are consistent with the limited existing evidence supporting the beneficial effects of behavioral weight loss interventions promoting nutritional education and behavior change on dietary behavior, diet quality, and/or adherence to the Mediterranean diet [29,30,31,32,33]. Patnode et al. [29], in a systematic review for the U.S. Preventive Services Task Force, found that healthful diet interventions in adults without known cardiovascular disease risk factors were related to reduced total energy and saturated fat intake and increased fiber and fruits and vegetables consumption. Similarly, a systematic review by Lin et al. [30,31], based on a large body of evidence (76 trials), also determined that an intensive combined lifestyle counseling significantly improved dietary behavior in participants with cardiovascular disease risk factors.

Studies exploring the effects of these dietary and/or lifestyle approaches on dietary patterns and quality of the diet in adults with moderate-to-severe OSA are currently lacking. Most previous studies in this regard only included calorie-restricted diets [25], which, as shown by the Look AHEAD (Action for Health in Diabetes) study [49]—the largest randomized trial in this field of research — may not be the most-efficient approach for diet quality and sustainable dietary behavior change [26,27]. According to corroborative evidence, caloric restriction may result in compensatory changes that cause increased hunger and, in turn, increased energy intake as a homeostatic response to fat loss [26,50]. Furthermore, caloric restriction has also been associated with a compensatory reduction in energy expenditure that prevents weight loss in the long term [50]. Most importantly, this approach is not focused on changing dietary patterns and diet quality in the long term, which are the key factors for weight loss and benefits maintenance.

The current study is the first to report the beneficial effects of an interdisciplinary weight loss and lifestyle intervention on dietary behavior and the association of these changes with changes in OSA severity and related outcomes in adults with moderate-to-severe OSA. Remarkably, those participants who achieved healthier dietary behavior and greater adherence to the Mediterranean diet also exhibited lower OSA severity, greater weight loss, and enhanced body composition and anthropometric parameters. Therefore, nutritional education and behavior change, focusing on macronutrients intake and promoting the Mediterranean diet, is an important intervention component to be considered and included in weight loss and lifestyle interventions for the management of OSA. The Mediterranean diet has been shown to have greater long-term beneficial effects on weight status and waist circumference than low-fat and calorie-restricted diets among individuals with type 2 diabetes and other cardiovascular risk factors [51]. In adults with OSA, the Mediterranean diet has also been shown to be related with reductions in body weight and abdominal fat, which is associated with reductions in OSA severity [28]. Furthermore, given its anti-inflammatory and antioxidant properties, the Mediterranean diet may potentially combat the inflammation and oxidative stress found in OSA, thereby improving the upper-airway neuromuscular control and muscle force-generating capacity and thus preventing the occurrence of the upper-airway obstructions during sleep [26].

The main strength of the current study is the design and implementation of a novel interdisciplinary weight loss and lifestyle intervention readily adaptable to real-world clinical practice. Another notable strength is the measurement of sleep and OSA severity through a full-night in-laboratory polysomnography—the gold-standard for the measurement of these outcomes—at each study assessment (baseline, intervention endpoint, and 6 months after intervention). Nevertheless, a limitation of the study design is the sole inclusion of men with moderate-to-severe OSA and overweight/obesity in our sample, which restricts the generalization of our findings. Furthermore, the study duration may have not been sufficient to determine long-term intervention effects and benefits maintenance. Another limitation is the subjective assessment of dietary behavior and adherence to the Mediterranean diet through the FBC and MEDAS, which, although widely used, are self-reported questionnaires. Therefore, future well-designed studies, including women, longer-term follow-ups, and objective dietary behavior assessments are needed.

## 5. Conclusions

In conclusion, in this study involving men with moderate-to-severe OSA and overweight/obesity, an eight-week interdisciplinary weight loss and lifestyle intervention, including dietary behavior change, moderate-intensity aerobic exercise, sleep hygiene, and tobacco and alcohol avoidance, was related to significant improvements in dietary behavior and adherence to the Mediterranean diet. Given the beneficial effects of dietary behavior change interventions and the Mediterranean diet on weight loss and OSA severity, approaches including these dietary components should be the strategy of choice for the comprehensive management of this increasingly common sleep-disordered breathing.

## Figures and Tables

**Table 1:** Baseline characteristics of the study participants.

	No. (%) ^a^	
Characteristics ^b^	Control (*n* = 49)	Intervention (*n* = 40)
Age, mean (SD), y	55.3 (8.5)	52.6 (7.1)
Sex, % Men	49 (100)	40 (100)
Educational level		
Primary Education	13 (26.5)	10 (25.0)
Secondary Education	10 (20.4)	6 (15.0)
Vocational Education	13 (26.5)	17 (42.5)
Higher Education	13 (26.5)	7 (17.5)
Marital status		
Single	7 (14.3)	2 (5.0)
Married	34 (69.4)	34 (85.0)
Divorced	8 (16.3)	4 (10.0)
Occupational status		
Employed	27 (55.1)	21 (52.5)
Self-employed	8 (16.3)	12 (30.0)
Unemployed	4 (8.2)	5 (12.5)
Retired	10 (20.4)	2 (5.0)
Medical Conditions ^c^		
Hypertension	33 (67.4)	27 (67.5)
Diabetes Mellitus II	12 (24.5)	10 (25.0)
Cardiovascular disease	9 (18.4)	7 (17.5)
Other medical conditions	29 (59.2)	26 (65.0)
Medication ^c^		
Antihypertensive	31 (63.3)	24 (60.0)
Statins	15 (30.6)	7 (17.5)
Oral antidiabetic	5 (10.2)	2 (5.0)
Insulin	3 (6.1)	1 (2.5)
Beta-blockers	7 (14.3)	5 (12.5)
Polymedication ^d^	14 (28.6)	6 (15.0)
Body mass index, mean (SD), kg/m^2^	33.9 (4.8)	35.0 (6.0)
Body weight, mean (SD), kg	99.6 (18.3)	103.3 (17.5)
Fat mass, mean (SD), kg	33.8 (9.0)	34.9 (10.6)
Visceral adipose tissue, mean (SD), g	1049.2 (260.3)	1017.3 (285.2)
Neck circumference, mean (SD), cm	45.5 (3.9)	45.0 (3.8)
Chest circumference, mean (SD), cm	117.4 (9.9)	118.0 (10.3)
Waist circumference, mean (SD), cm	117.9 (12.2)	119.0 (12.4)
Apnea-hypopnea index, mean (SD), events/h	41.1 (21.3)	41.6 (23.5)
Obstructive sleep apnea severity		
Moderate	20 (40.8)	15 (37.5)
Severe	29 (59.2)	25 (62.5)
Oxygen desaturation index, mean (SD), events/h	45.4 (21.1)	45.4 (27.7)
Sleep efficiency, mean (SD), %	85.6 (8.1)	86.0 (9.1)

^a^ Results are presented as mean (standard deviation) for normally distributed numerical variables and as absolute number (relative frequency) for categorical variables. ^b^ No significant between-group differences were observed in any of the baseline characteristics. ^c^ Participants could have more than one condition or medication. ^d^ Defined as the use of five or more medications.

**Table 2:** Dietary behavior outcomes.

Outcomes	Control (*n* = 49)		Intervention (*n* = 40)		Difference between Groups, Mean (95% CI) ^a^
	Mean (95% CI)	Change from Baseline, Mean (95% CI)	Mean (95% CI)	Change from Baseline, Mean (95% CI)	
**Food Behavior Checklist, total score ^d^**					
At baseline	59.1 (56.8 to 61.4)		59.5 (56.9 to 62.0)		
At 8 weeks	62.4 (59.9 to 64.9)	3.3 (0.6 to 5.9)	71.5 (68.9 to 74.0)	12.0 (9.5 to 14.5)	8.7 (5.7 to 11.7) ^c^
At 6 months	60.6 (57.9 to 63.4)	1.5 (−1.4 to 4.4)	68.6 (66.0 to 71.3)	9.2 (6.5 to 11.9)	7.7 (4.4 to 10.9) ^c^
** Fruit and vegetables consumption score**					
At baseline	21.5 (20.0 to 22.9)		22.6 (21.0 to 24.2)		
At 8 weeks	23.1 (21.5 to 24.7)	1.6 (−0.2 to 3.4)	29.5 (27.9 to 31.1)	6.9 (5.2 to 8.6)	5.2 (3.2 to 7.3) ^c^
At 6 months	22.4 (20.7 to 24.2)	0.9 (−1.0 to 2.9)	27.5 (25.8 to 29.1)	4.9 (3.0 to 6.7)	3.9 (1.7 to 6.1) ^c^
** Milk/dairy consumption score**					
At baseline	6.0 (5.5 to 6.4)		5.8 (5.3 to 6.3)		
At 8 weeks	5.6 (5.1 to 6.2)	−0.3 (−0.9 to 0.2)	5.7 (5.2 to 6.2)	−0.1 (−0.6 to 0.4)	0.2 (−0.4 to 0.8)
At 6 months	5.9 (5.3 to 6.4)	−0.1 (−0.7 to 0.5)	5.4 (4.9 to 6.0)	−0.4 (−0.9 to 0.2)	−0.3 (−1.0 to 0.4)
** Food security score**					
At baseline	3.1 (2.9 to 3.4)		3.1 (2.8 to 3.3)		
At 8 weeks	3.3 (3.0 to 3.5)	0.1 (−0.2 to 0.4)	3.1 (2.8 to 3.4)	0.1 (−0.2 to 0.4)	−0.1 (−0.4 to 0.3)
At 6 months	3.2 (3.0 to 3.5)	0.1 (−0.2 to 0.4)	3.2 (2.9 to 3.5)	0.2 (−0.2 to 0.5)	0.1 (−0.3 to 0.5)
** Diet quality score**					
At baseline	9.9 (9.3 to 10.5)		9.7 (9.0 to 10.4)		
At 8 weeks	10.5 (9.9 to 11.2)	0.7 (−0.1 to 1.4)	12.2 (11.5 to 12.8)	2.5 (1.8 to 3.2)	1.8 (1.0 to 2.6) ^c^
At 6 months	10.3 (9.5 to 11.0)	0.4 (−0.4 to 1.2)	11.8 (11.1 to 12.5)	2.1 (1.4 to 2.9)	1.7 (0.8 to 2.6) ^c^
** Fast food consumption score**					
At baseline	7.4 (7.0 to 7.9)		7.2 (6.7 to 7.7)		
At 8 weeks	8.2 (7.6 to 8.8)	0.8 (−0.1 to 1.6)	8.6 (8.1 to 9.1)	1.4 (0.6 to 2.2)	0.6 (−0.3 to 1.6)
At 6 months	7.7 (7.1 to 8.4)	0.3 (−0.6 to 1.2)	8.3 (7.8 to 8.9)	1.1 (0.3 to 2.0)	0.8 (−0.2 to 1.9)
** Sweetened beverages consumption score**					
At baseline	6.7 (6.5 to 7.0)		6.6 (6.3 to 6.9)		
At 8 weeks	7.1 (6.8 to 7.4)	0.3 (−0.1 to 0.7)	7.5 (7.2 to 7.8)	0.9 (0.5 to 1.3)	0.5 (0.1 to 1.0) ^b^
At 6 months	6.9 (6.5 to 7.2)	0.1 (−0.3 to 0.6)	7.4 (7.1 to 7.7)	0.7 (0.3 to 1.1)	0.6 (0.1 to 1.1) ^b^
** Meat consumption score**					
At baseline	1.9 (1.6 to 2.1)		1.9 (1.6 to 2.2)		
At 8 weeks	2.1 (1.8 to 2.5)	0.3 (−0.2 to 0.8)	2.3 (2.0 to 2.6)	0.4 (−0.1 to 0.9)	0.1 (−0.5 to 0.7)
At 6 months	2.0 (1.7 to 2.4)	0.2 (−0.4 to 0.7)	2.5 (2.1 to 2.8)	0.6 (0.1 to 1.1)	0.4 (−0.2 to 1.0)
**Mediterranean Diet Adherence Screener, total score ^e^**					
At baseline	8.2 (7.7 to 8.7)		8.1 (7.6 to 8.6)		
At 8 weeks	8.8 (8.2 to 9.4)	0.6 (−0.1 to 1.3)	10.7 (10.2 to 11.3)	2.7 (1.9 to 3.3)	2.1 (1.3 to 2.9) ^c^
At 6 months	8.5 (7.9 to 9.1)	0.3 (−0.5 to 1.1)	10.1 (9.6 to 10.7)	2.0 (1.3 to 2.8)	1.7 (0.9 to 2.6) ^c^

Abbreviations: CI, confidence interval. ^a^ Using the group × visit interaction term from a linear mixed-effects model, including study group, time (baseline, 8 weeks and 6 months), and study group × time as fixed effects and participant as random effects. ^b^ *p* < 0.05 from the time × study group interactions. ^c^ *p* < 0.001 from the time × study group interactions. ^d^ The Food Behavior Checklist assesses dietary behavior (range, 23–85; higher scores indicate healthier dietary behavior) [45]. ^e^ The Mediterranean Diet Adherence Screener assesses adherence to the Mediterranean diet (range, 0–14; higher scores indicate greater adherence; scores ≥ 10 indicate high adherence to the Mediterranean diet) [46].

**Table 3:** Repeated measures correlation analyses examining the association of changes in dietary behavior over time with changes in sleep and body weight and composition outcomes.

Outcomes	Food Behavior Checklist, Total Score ^a^			Mediterranean Diet Adherence Screener, Total Score ^b^		
	r	95% CI	*p* Value	r	95% CI	*p* Value
**Changes at 8 weeks and 6 months after intervention**						
Apnea-hypopnea index, events/h	−0.71	−0.81 to −0.58	< 0.001	−0.66	−0.77 to −0.51	<0.001
Oxygen desaturation index ≥ 3%, events/h	−0.58	−0.71 to −0.40	<0.001	−0.51	−0.66 to −0.31	<0.001
Sleep efficiency, %	0.36	0.15 to 0.55	0.001	0.43	0.22 to 0.60	<0.001
Body weight, kg	−0.65	−0.77 to −0.49	<0.001	−0.65	−0.76 to −0.49	<0.001
Fat mass, kg	−0.44	−0.61 to −0.23	<0.001	−0.47	−0.63 to −0.27	<0.001
Visceral adipose tissue, g	−0.42	−0.59 to −0.21	<0.001	−0.40	−0.58 to −0.19	<0.001
Neck circumference, cm	−0.64	−0.76 to −0.48	<0.001	−0.67	−0.78 to −0.52	<0.001
Chest circumference, cm	−0.53	−0.67 to −0.34	<0.001	−0.54	−0.69 to −0.36	<0.001
Waist circumference, cm	−0.65	−0.76 to −0.49	<0.001	−0.65	−0.77 to −0.50	<0.001
**Changes from baseline to 8 weeks**						
Apnea-hypopnea index, events/h	−0.79	−0.88 to −0.63	<0.001	−0.79	−0.88 to −0.63	<0.001
Oxygen desaturation index ≥ 3%, events/h	−0.66	−0.81 to −0.43	<0.001	−0.63	−0.79 to −0.39	<0.001
Sleep efficiency, %	0.50	0.22 to 0.70	<0.001	0.49	−0.20 to 0.70	0.001
Body weight, kg	−0.79	−0.89 to −0.63	<0.001	−0.78	−0.88 to −0.62	<0.001
Fat mass, kg	−0.50	−0.71 to −0.22	<0.001	−0.52	−0.72 to −0.24	<0.001
Visceral adipose tissue, g	−0.47	−0.68 to −0.18	0.002	−0.44	−0.66 to −0.14	0.004
Neck circumference, cm	−0.74	−0.86 to −0.56	<0.001	−0.78	−0.88 to −0.61	<0.001
Chest circumference, cm	−0.64	−0.79 to −0.40	<0.001	−0.63	−0.79 to −0.39	<0.001
Waist circumference, cm	−0.75	−0.86 to −0.57	<0.001	−0.76	−0.87 to −0.59	<0.001
**Changes from baseline to 6 months after intervention**						
Apnea-hypopnea index, events/h	−0.78	−0.89 to −0.60	<0.001	−0.67	−0.82 to −0.42	<0.001
Oxygen desaturation index ≥ 3%, events/h	−0.73	−0.86 to −0.52	<0.001	−0.58	−0.77 to −0.30	<0.001
Sleep efficiency, %	0.38	0.05 to 0.64	0.02	0.49	0.18 to 0.71	0.003
Body weight, kg	−0.70	−0.84 to −0.46	<0.001	−0.68	−0.83 to −0.44	<0.001
Fat mass, kg	−0.72	−0.85 to −0.50	<0.001	−0.69	−0.83 to −0.45	<0.001
Visceral adipose tissue, g	−0.73	−0.86 to −0.51	<0.001	−0.69	−0.83 to −0.45	<0.001
Neck circumference, cm	−0.70	−0.84 to −0.48	<0.001	−0.72	−0.85 to −0.49	<0.001
Chest circumference, cm	−0.54	−0.75 to −0.25	<0.001	−0.57	−0.77 to −0.29	<0.001
Waist circumference, cm	−0.74	−0.86 to −0.52	<0.001	−0.71	−0.85 to −0.49	<0.001

Abbreviations: CI, confidence interval. ^a^ The Food Behavior Checklist assesses dietary behavior (range, 23–85; higher scores indicate healthier dietary behavior) [45]. ^b^ The Mediterranean Diet Adherence Screener assesses adherence to the Mediterranean diet (range, 0–14; higher scores indicate greater adherence; scores ≥ 10 indicate high adherence to the Mediterranean diet) [46].

## Data Availability

Requests for data can be made to A.C.-B. (acarneiro@ugr.es). Data, code book, and analytic code will be made available upon request subject to team review and approval of the proposed analysis.

## Supplementary Materials

The following supporting information can be downloaded at: https://www.mdpi.com/article/10.3390/nu14132731/s1, Table S1: Dietary behavior outcomes (Per protocol); Table S2: Dietary behavior outcomes (change from 8 weeks to 6 months after intervention); Figure S1: Dietary behavior outcomes (change from 8 weeks to 6 months after intervention).
