# Supplementary material for: Effect of a Weight Loss and Lifestyle Intervention on Dietary Behavior in Men with Obstructive Sleep Apnea: The INTERAPNEA Trial
Source: Nutrients. 2022 Jun 30;14(13):2731. doi: 10.3390/nu14132731 (PMC9268365; doi:10.3390/nu14132731)
Supplement: Supplementary file 1 [file nutrients-14-02731-s001.zip › nutrients-1789498-supplementary.pdf]

SUPPLEMENTARY MATERIAL

Effect of a weight loss and lifestyle intervention on dietary behavior in men with obstructive sleep apnea: The INTERAPNEA trial

Table S1. Dietary behavior outcomes (Per protocol)

	Control (n=35)		Intervention (n=40)		Difference between groups, mean (95% CI)[a]
	Mean (95% CI)[a]	Change from baseline, mean (95% CI)	Mean (95% CI)[a]	Change from baseline, mean (95% CI)	
**Food Behavior Checklist, total score[c]**					
At baseline	59.6 (57.0 to 62.3)		59.5 (57.0 to 62.0)		
At 8 weeks	62.8 (60.1 to 65.5)	3.1 (0.4 to 5.9)	71.5 (69.0 to 74.0)	12.0 (9.5 to 14.5)	8.9 (5.8 to 11.9)[b]
At 6 months	61.1 (58.2 to 64.0)	1.5 (-1.5 to 4.5)	68.6 (66.1 to 71.2)	9.2 (6.5 to 11.9)	7.7 (4.4 to 11.0)[b]
**Fruit and vegetables consumption score**					
At baseline	21.8 (20.0 to 22.9)		22.6 (21.0 to 24.2)		
At 8 weeks	23.3 (21.5 to 24.7)	1.5 (-0.4 to 3.3)	29.5 (27.8 to 31.1)	6.9 (5.1 to 8.6)	5.4 (3.3 to 7.4)[b]
At 6 months	22.7 (20.7 to 24.2)	0.8 (-1.2 to 2.9)	27.5 (25.8 to 29.2)	4.9 (3.0 to 6.7)	4.0 (1.8 to 6.3)[b]
**Milk/dairy consumption score**					
At baseline	6.1 (5.5 to 6.6)		5.8 (5.3 to 6.3)		
At 8 weeks	5.7 (5.2 to 6.3)	-0.3 (-0.9 to 0.2)	5.7 (5.2 to 6.2)	-0.1 (-0.6 to 0.4)	0.2 (-0.4 to 0.8)
At 6 months	6.0 (5.4 to 6.6)	-0.1 (-0.7 to 0.5)	5.4 (4.9 to 5.9)	-0.4 (-0.9 to 0.2)	-0.3 (-1.0 to 0.4)
**Food security score**					
At baseline	3.1 (2.9 to 3.4)		3.1 (2.8 to 3.3)		
At 8 weeks	3.3 (3.0 to 3.5)	0.1 (-0.2 to 0.4)	3.1 (2.9 to 3.4)	0.1 (-0.2 to 0.4)	-0.03 (-0.4 to 0.3)
At 6 months	3.2 (2.9 to 3.5)	0.1 (-0.3 to 0.4)	3.2 (2.9 to 3.5)	0.2 (-0.2 to 0.5)	0.1 (-0.3 to 0.5)
**Diet quality score**					
At baseline	9.8 (9.1 to 10.5)		9.7 (9.0 to 10.3)		
At 8 weeks	10.5 (9.8 to 11.2)	0.7 (-0.03 to 1.5)	12.2 (11.5 to 12.8)	2.5 (1.8 to 3.2)	1.8 (0.9 to 2.6)[b]
At 6 months	10.3 (9.5 to 11.0)	0.5 (-0.3 to 1.3)	11.8 (11.1 to 12.5)	2.1 (1.4 to 2.9)	1.6 (0.7 to 2.6)[b]
**Fast food consumption score**					
At baseline	7.7 (7.1 to 8.2)		7.2 (6.7 to 7.7)		
At 8 weeks	8.3 (7.7 to 8.8)	0.6 (-0.3 to 1.4)	8.6 (8.1 to 9.1)	1.4 (0.6 to 2.2)	0.8 (-0.1 to 1.8)
At 6 months	7.9 (7.2 to 8.5)	0.2 (-0.8 to 1.1)	8.3 (7.8 to 8.9)	1.1 (0.3 to 2.0)	1.0 (-0.1 to 2.0)
**Sweetened beverages consumption score**					
At baseline	6.7 (6.5 to 7.0)		6.6 (6.3 to 6.9)		
At 8 weeks	7.1 (6.8 to 7.4)	0.4 (0.002 to 0.9)	7.5 (7.2 to 7.8)	0.9 (0.5 to 1.3)	0.4 (-0.03 to 0.9)
At 6 months	6.9 (6.5 to 7.2)	0.2 (-0.2 to 0.7)	7.4 (7.1 to 7.7)	0.7 (0.3 to 1.1)	0.5 (-0.02 to 1.0)
**Meat consumption score**					
At baseline	1.9 (1.6 to 2.1)		1.9 (1.6 to 2.2)		
At 8 weeks	2.1 (1.8 to 2.5)	0.2 (-0.3 to 0.8)	2.3 (2.0 to 2.6)	0.4 (-0.1 to 0.9)	0.2 (-0.4 to 0.8)
At 6 months	2.0 (1.7 to 2.4)	0.2 (-0.4 to 0.8)	2.5 (2.1 to 2.8)	0.6 (0.1 to 1.1)	0.4 (-0.2 to 1.1)
**Mediterranean Diet Adherence Screener, total score[d]**					
At baseline	8.1 (7.6 to 8.7)		8.1 (7.6 to 8.6)		
At 8 weeks	8.7 (8.2 to 9.3)	0.6 (-0.1 to 1.3)	10.7 (10.2 to 11.3)	2.6 (1.9 to 3.3)	2.0 (1.2 to 2.9)[b]
At 6 months	8.5 (7.8 to 9.1)	0.3 (-0.5 to 1.1)	10.1 (9.6 to 10.7)	2.0 (1.3 to 2.8)	1.7 (0.8 to 2.6)[b]

Abbreviations: CI, confidence interval.

[a] Using the group × visit interaction term from a linear mixed-effects model including study group, time (baseline, 8 weeks and 6 months), and study group × time as fixed effects and participant as random effects.

[b] $P < 0.001$ from the time × study group interactions.

[c] The Food Behavior Checklist assesses dietary behavior (range, 23-85; higher scores indicate healthier dietary behavior).

[d] The Mediterranean Diet Adherence Screener assesses adherence to the Mediterranean diet (range, 0-14; higher scores indicate greater adherence; scores ≥10 indicate high adherence to the Mediterranean diet).

Table S2. Dietary behavior outcomes (Change from 8 weeks to 6 months after intervention)

	Control				Intervention			
	N	8 weeks Mean (95% CI)	6 months Mean (95% CI)	Mean change (95% CI)[a]	N	8 weeks Mean (95% CI)	6 months Mean (95% CI)	Mean change (95% CI)[a]
**Intention-to-treat approach**								
Food Behavior Checklist[c]								
Total score	49	62.4 (59.9 to 64.9)	60.6 (57.9 to 63.4)	-1.8 (-4.8 to 1.2)	40	71.5 (68.9 to 74.0)	68.6 (66.0 to 71.3)	-2.8 (-5.5 to -0.1)[b]
Fruit and vegetables consumption score	49	23.1 (21.5 to 24.7)	22.4 (20.7 to 24.2)	-0.7 (-2.7 to 1.3)	40	29.5 (27.9 to 31.1)	27.5 (25.8 to 29.1)	-2.0 (-3.8 to -0.2)[b]
Milk/dairy consumption score	49	5.6 (5.1 to 6.2)	5.9 (5.3 to 6.4)	0.2 (-0.4 to 0.8)	40	5.7 (5.2 to 6.2)	5.4 (4.9 to 6.0)	-0.3 (-0.8 to 0.3)
Food security score	49	3.3 (3.0 to 3.5)	3.2 (3.0 to 3.5)	-0.03 (-0.4 to 0.3)	40	3.1 (2.8 to 3.4)	3.2 (2.9 to 3.5)	0.1 (-0.2 to 0.4)
Diet quality score	49	10.5 (9.9 to 11.2)	10.3 (9.5 to 11.0)	-0.3 (-1.1 to 0.6)	40	12.2 (11.5 to 12.8)	11.8 (11.1 to 12.5)	-0.3 (-1.1 to 0.4)
Fast food consumption score	49	8.2 (7.6 to 8.8)	7.7 (7.1 to 8.4)	-0.5 (-1.4 to 0.5)	40	8.6 (8.1 to 9.1)	8.3 (7.8 to 8.9)	-0.3 (-1.1 to 0.6)
Sweetened beverages consumption score	49	7.1 (6.8 to 7.4)	6.9 (6.5 to 7.2)	-0.2 (-0.7 to 0.3)	40	7.5 (7.2 to 7.8)	7.4 (7.1 to 7.7)	-0.1 (-0.6 to 0.3)
Meat consumption score	49	2.1 (1.8 to 2.5)	2.0 (1.7 to 2.4)	-0.1 (-0.7 to 0.5)	40	2.3 (2.0 to 2.6)	2.5 (2.1 to 2.8)	0.2 (-0.3 to 0.7)
Mediterranean Diet Adherence Screener[d]								
Total score	49	8.8 (8.2 to 9.4)	8.5 (7.9 to 9.1)	-0.3 (-1.1 to 0.5)	40	10.7 (10.2 to 11.3)	10.1 (9.6 to 10.7)	-0.6 (-1.3 to 0.1)
**Per-protocol approach**								
Food Behavior Checklist[c]								
Total score	49	62.8 (60.1 to 65.5)	61.1 (58.2 to 64.0)	-1.7 (-4.7 to 1.4)	40	71.5 (68.9 to 74.0)	68.6 (66.0 to 71.3)	-2.8 (-5.5 to -0.1)[b]
Fruit and vegetables consumption score	49	23.3 (21.5 to 24.7)	22.7 (20.7 to 24.2)	-0.7 (-2.7 to 1.4)	40	29.5 (27.9 to 31.1)	27.5 (25.8 to 29.1)	-2.0 (-3.8 to -0.2)[b]
Milk/dairy consumption score	49	5.7 (5.2 to 6.3)	6.0 (5.4 to 6.6)	0.3 (-0.4 to 0.9)	40	5.7 (5.2 to 6.2)	5.4 (4.9 to 6.0)	-0.3 (-0.8 to 0.3)
Food security score	49	3.3 (3.0 to 3.5)	3.2 (2.9 to 3.5)	-0.1 (-0.4 to 0.3)	40	3.1 (2.8 to 3.4)	3.2 (2.9 to 3.5)	0.1 (-0.2 to 0.4)
Diet quality score	49	10.5 (9.8 to 11.2)	10.3 (9.5 to 11.0)	-0.2 (-1.1 to 0.6)	40	12.2 (11.5 to 12.8)	11.8 (11.1 to 12.5)	-0.3 (-1.1 to 0.4)
Fast food consumption score	49	8.3 (7.7 to 8.8)	7.9 (7.2 to 8.5)	-0.4 (-1.3 to 0.6)	40	8.6 (8.1 to 9.1)	8.3 (7.8 to 8.9)	-0.3 (-1.1 to 0.6)
Sweetened beverages consumption score	49	7.1 (6.8 to 7.4)	6.9 (6.5 to 7.2)	-0.2 (-0.7 to 0.3)	40	7.5 (7.2 to 7.8)	7.4 (7.1 to 7.7)	-0.1 (-0.6 to 0.3)
Meat consumption score	49	2.1 (1.8 to 2.5)	2.0 (1.7 to 2.4)	-0.1 (-0.6 to 0.5)	40	2.3 (2.0 to 2.6)	2.5 (2.1 to 2.8)	0.2 (-0.3 to 0.7)
Mediterranean Diet Adherence Screener[d]								
Total score	49	8.7 (8.2 to 9.3)	8.5 (7.8 to 9.1)	-0.3 (-1.1 to 0.5)	40	10.7 (10.2 to 11.3)	10.1 (9.6 to 10.7)	-0.6 (-1.3 to 0.1)

Abbreviations: CI, confidence interval.

[a] Using post-hoc test (pairwise comparison) in a linear mixed-effects model including study group, time (baseline, 8 weeks and 6 months), and study group × time as fixed effects and participant as random effects.

[b] $P < 0.05$.

[c] The Food Behavior Checklist assesses dietary behavior (range, 23-85; higher scores indicate healthier dietary behavior).

[d] The Mediterranean Diet Adherence Screener assesses adherence to the Mediterranean diet (range, 0-14; higher scores indicate greater adherence; scores ≥10 indicate high adherence to the Mediterranean diet).

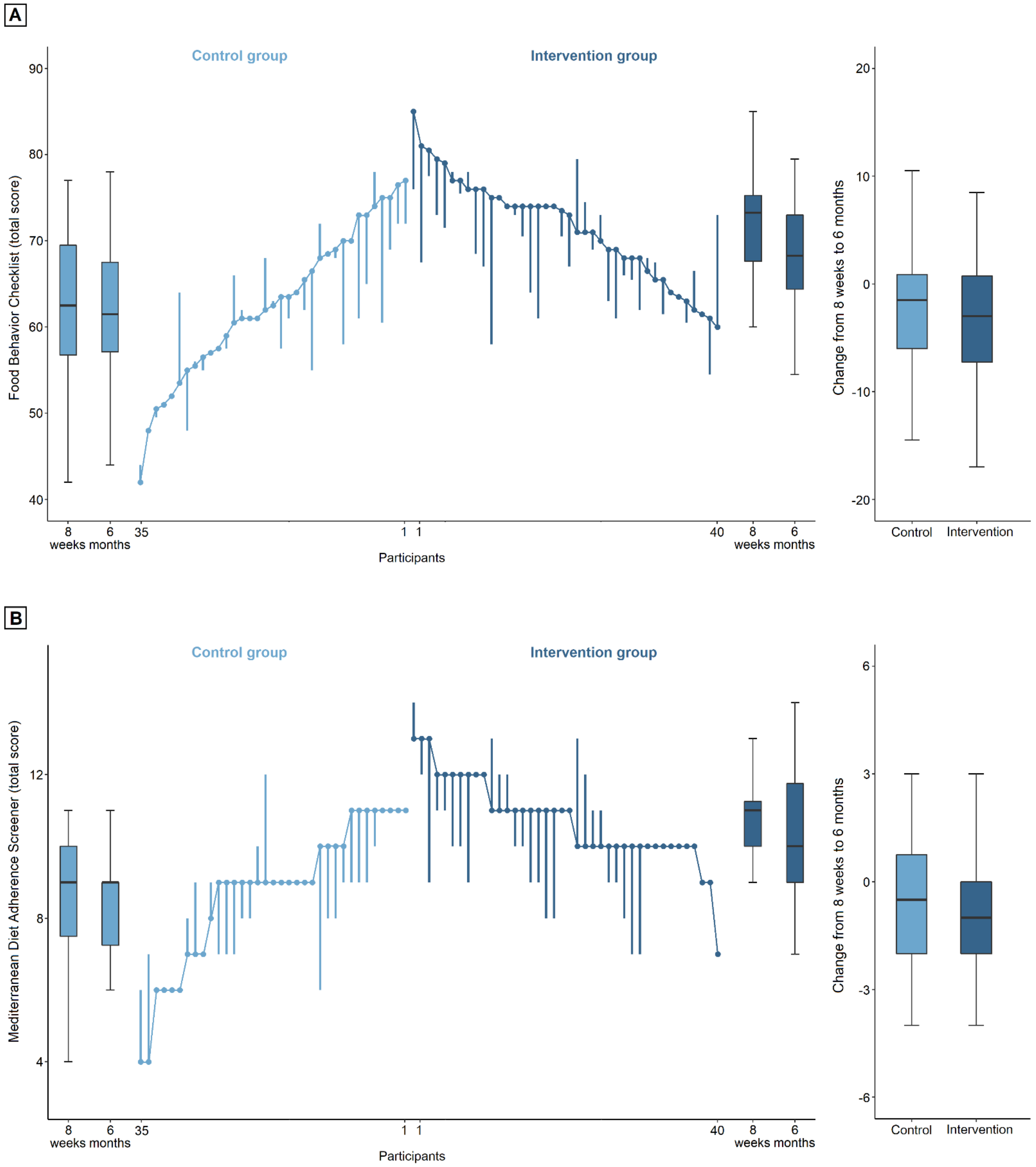

Figure S1. Dietary Behavior Outcomes (Change from 8 weeks to 6 months after intervention). The ends of the boxes in the boxplots are located at the first and third quartiles, with the black line in the middle illustrating the median. Whiskers extend to the upper and lower adjacent values, the location of the furthest point within a distance of 1.5 interquartile ranges from the first and third quartiles. The parallel line plot contains 1 vertical line for each patient which extends from their 8-week value to their 6-month value. Ascending lines indicate an improvement in the outcome. Eight-week values are placed in ascending order for the control group and descending order for the intervention group. A, The Food Behavior Checklist assesses dietary behavior (range, 23-85; higher scores indicate healthier dietary behavior). B, The Mediterranean Diet Adherence Screener assesses adherence to the Mediterranean diet (range, 0-14; higher scores indicate greater adherence; scores ≥10 indicate high adherence to the Mediterranean diet).
